# Biological and genomic characterization of three psychrophilic *Y. enterocolitica* phages

**DOI:** 10.3389/fmicb.2024.1423610

**Published:** 2024-07-11

**Authors:** Jens A. Hammerl, Minh Anh Pham, Shirin El-Ahmad, Diana Manta, Claudia Jäckel, Stefan Hertwig

**Affiliations:** Department of Biological Safety, German Federal Institute for Risk Assessment, Berlin, Germany

**Keywords:** *Yersinia*, phages, application, genome, temperature

## Abstract

*Yersinia* (*Y.*) *enterocolitica* is an important foodborne pathogenic species that is mainly transmitted by the consumption of contaminated meat, particularly pork. To combat the bacteria along the food chain, the application of strictly lytic phages may be a promising tool. As the temperatures in the gut of animals and during food processing can differ significantly, a phage cocktail intended to be used for applications should comprise phages that are active at various temperatures. In this study, we isolated and characterized three phages with a myoviridal morphology (vB_YenM_P8, vB_YenM_P744 and vB_YenM_P778), which lysed the most important *Y. enterocolitica* serotypes O:3, O:9 and O:5,27 at a low multiplicity of infection (MOI) and at low temperatures down to 6°C. While vB_YenM_P8 is a member of the T4 family, vB_YenM_P744 and vB_YenM_P778 are novel phages that do not show relationship to known phages. The three phages were mixed in a cocktail with the already described phages vB_YenM_P281 and vB_YenP_Rambo. The cocktail revealed a strong lytic activity and lysed a mixture of *Y. enterocolitica* serotypes at room temperature (RT) within few hours with a reduction of up to 4.8 log_10_ units. Moreover, at even lower temperatures the mixture was significantly reduced after incubation overnight. The strongest reductions were determined at 6°C (4.0 log_10_ units) suggesting that the cocktail can lyse the psychrophilic *Y. enterocolitica* also during food processing. To determine possible phage resistance, 100 colonies that survived the infection by the phages were isolated and analysed regarding their serotype and phage susceptibility. Most isolates belonged to serotype O:9, but all of them were still sensitive to at least one phage of the cocktail.

## Introduction

The genus *Yersinia* contains 28 species, three of which are pathogenic for humans (access: January 2024).^1^ *Yersinia* (*Y*.) *pestis* is the causative agent of plague, transmitted by the bite of an infected flea. On the other hand, *Y. pseudotuberculosis* and particularly *Y. enterocolitica* are foodborne enteropathogenic species, which may cause gastrointestinal diseases termed yersiniosis (Le Guern et al., 2016). In 2022, yersiniosis was the third most commonly reported bacterial foodborne zoonotic disease in the European Union (EU) (European Food Safety Authority and European Centre for Disease Prevention and Control, 2023). Typical symptoms are fever, abdominal pain and diarrhea (Bancerz-Kisiel and Szweda, 2015). While human yersiniosis is mainly caused by *Y. enterocolitica*, *Y. pseudotuberculosis* is more often found in wild and zoo animals causing various infections, some of which are lethal and exhibit tuberculosis-like symptoms. By contrast, in Europe and many other countries, the presence of *Y. enterocolitica* is strongly associated with domestic pigs, which do not get ill, but represent a natural reservoir for the bacteria (Fredriksson-Ahomaa et al., 2006a,b; Bancerz-Kisiel and Szweda, 2015; European Food Safety Authority, 2015). Human infections by this species are predominantly linked to the consumption of raw or insufficiently cooked pork (Laukkanen et al., 2008). However, raw milk, water and vegetables such as mixed salad contaminated with *Y. enterocolitica* have also been reported as possible sources of infection (Jamali et al., 2015; MacDonald et al., 2016; Terech-Majewska et al., 2016).

*Y. enterocolitica* is divided into six biotypes (1A, 1B, 2, 3, 4 and 5) and more than 70 serotypes (O) (Bancerz-Kisiel et al., 2018). In Europe, the most clinically important bioserotype is 4/O:3, followed by 2/O:9 and 2/O:5,27. Biotype 1A strains frequently occur in the environment and food and are generally regarded as nonpathogenic, although some reports have isolated this biotype from sick individuals (Joutsen et al., 2016; Bancerz-Kisiel et al., 2018).

Since the use of antibiotics is generally avoided in food production, alternative methods are needed to reduce these bacteria along the food chain. One alternative approach to treating bacterial infections or reducing pathogens in food is the application of virulent (strictly lytic) phages, which can also be effective against multidrug-resistant bacteria (Xu, 2021). A number of phages lysing *Y. enterocolitica* has been described, most of them are podoviruses and myoviruses (Salem et al., 2015; Gwak et al., 2018; Salem and Skurnik, 2018). Many podoviruses infecting *Y. enterocolitica* are related to T7 and have a rather narrow host range as they mainly lyse O:3 strains (Pajunen et al., 2001; Leon-Velarde et al., 2014; Liang et al., 2016). An exemption is the T7-like phage vB_YenP_Rambo that lysed 50 out of 62 and 54 out of 62 pathogenic *Y. enterocolitica* strains belonging to several bioserotypes at 28°C and 37°C, respectively (Hammerl et al., 2022b). *Y. enterocolitica* myoviruses mostly exhibited a broader host range than podoviruses, because they could additionally lyse strains belonging to the serotypes O:9 and O:5,27. One myovirus (fHe-Yen9-01) that lysed 61.3% of 106 tested *Yersinia* strains was used for reduction experiments with food samples (pork and milk) and kitchenware and was able to reduce an O:9 strain significantly (Jun et al., 2018). The widest host range of all *Yersinia* phages described thus far showed the myoviruses PY100 and the very similar vB_YenM_P281. These two phages not only infected various bioserotypes of *Y. enterocolitica* (61.1% of 72 strains at 28°C and 95.8% at 37°C) but also strains of *Y. pseudotuberculosis* if the medium was supplemented with calcium and magnesium cations (Hammerl et al., 2022b). A combination of vB_YenM_P281 and vB_YenP_Rambo reduced an O:3 and an O:9 strain efficiently down to a temperature of 17°C. Phage-induced lysis of *Enterobacteriaceae* at low temperatures generally occurs much more slowly than at higher temperatures, such as 37°C, due to the limited propagation of the host bacteria. Additionally, higher multiplicities of infection (MOIs) are often required to achieve strong lytic activity, as shown for the phages vB_YenM_P281 and vB_YenP_Rambo, where a tenfold higher MOI was required for a significant reduction of the tested strains at 17°C (Hammerl et al., 2022b). The access to host cell receptors, important for binding of a phage may also change with the temperature.

In this study, we isolated and characterized three novel virulent myoviruses from game animals (vB_YenM_P744 and vB_YenM_P778) and sewage (vB_YenM_P8). These phages demonstrated excellent lytic activity at low temperatures and low MOIs, complementing each other well regarding their host range. We further evaluated a cocktail of these three phages, together with vB_YenM_P281 and vB_YenP_Rambo, which efficiently lysed a mixture of pathogenic *Y. enterocolitica* strains.

## Materials and methods

### Bacterial strains and culture conditions

The strains used in this study originate from the culture collection of the Consiliary Laboratory for *Yersinia* (KL *Yersinia*) at the German Federal Institute for Risk Assessment (BfR), Berlin, Germany (Hammerl et al., 2021b). Bacteria were cultivated in/on lysogeny broth (LB)-based media at temperatures between 6°C and 37°C, as specified (Hammerl et al., 2008). Cultivation in liquid medium was conducted under continuous shaking at 200–225 rpm.

### Isolation, propagation, and purification of phages

Two phages (vB_YenM_P744 and vB_YenM_P778) described here were isolated from fecal samples of wild boars hunted in northeast Germany. Phage vB_YenM_P8 was isolated from a sewage sample. At the KL *Yersinia*, 5 mL of SM buffer (Jäckel et al., 2015) were added to suspend the fecal samples overnight at 4°C on a stirrer. Thereafter, the material was subjected to centrifugation for 20 min at 8,000 rpm and 10°C. The supernatants were passed successively through 0.45 and 0.22 μm pore-size filters (VWR International, Darmstadt, Germany) and stored until further usage at 4°C. The lytic activity of the phages was determined by spot assays. For this, 10 μL of serial dilutions of the samples were applied onto the lawn of *Y. enterocolitica* indicator strains belonging to various serotypes. After incubation overnight at RT, (20-22°C), 30°C and 37°C, agar plates were inspected for plaque formation. Single plaques were subjected to a three-fold plaque purification procedure. High-titer lysates of the phages were obtained by preparing 10–20 agar plates with confluent lysis of the host bacteria. The soft agar was harvested by scraping and resuspended in SM buffer for four hours. Thereafter, the lysates were centrifuged for 20 min at 10,000 × g and then filtered (see above). Phages were concentrated by ultracentrifugation and purified using CsCl step gradients as previously described (Jäckel et al., 2017).

### Determination of the host range and efficiency of plating (EOP)

The host range of the purified phages was determined by spot activity assays. For this, 100–200 μL of each indicator strain were mixed with 6 mL prewarmed NZCYM (VWR International, Darmstadt, Germany) soft agar (0.6%) and poured onto a LB agar plate (Jäckel et al., 2015). Ten microliters of serial dilutions of each lysate (adjusted to ∼1 × 10^7^ pfu/mL) were spotted onto the overlay agar and lytic activity was visually inspected after an incubation overnight at RT, 30°C or 37°C. Further information on the tested strains is given by Hammerl et al. (2022a,b).

### Determination of the lytic activity of the phages at different temperatures

For this set of experiments, the well characterized DSMZ reference strains DSM 9676 (4/O:3) isolated from a mesenteric lymphnode in Sweden, DSM 11504 (2/O:5,27, country of origin unknown), DSM 11503 (2/O:9) isolated from a clinical specimen in Germany and the 2/O:9 strain Evira 663 isolated from a pig carcass swab in Belgium, 2011 (Hallanvuo et al., 2019), were used. Reduction of single strains was determined as follows: An overnight culture of each tested strain was transferred to fresh 10 mL LB broth and grown at different temperatures (6°C to RT) to an OD_588_ of 0.2, corresponding to approximately 1 x 10^8^ CFU/mL, determined by plating. The culture was then divided into two equal portions. One sample was infected with phage at a MOI of 0.1 (∼10^8^ PFU), the other was used as a control without added phages. The OD588 values of the cultures were measured every 30 minutes to monitor bacterial growth, especially important due to the slow growth at low temperatures. To determine cell numbers at the end of the experiment, 100 μl of a serial dilution of the control and phage-treated culture were plated on LB agar plates that were incubated overnight at 28°C. The following day, colonies were counted and the difference between the controls and phage-treated cultures was calculated. The reduction of a mixture of strains belonging to the serotype O:3, O:9 and O:5,27 was performed by incubating each strain up to an OD_588_ of approximately 0.2. Thereafter, the strains were mixed together. However, here the three phages vB_YenM_P744, vB_YenM_P778 and vB_YenM_P8 were used in a cocktail in combination with vB_YenM_P281 and vB_YenP_Rambo, which were previously described (Hammerl et al., 2021b). To obtain a total MOI of 0.1, each of the five phages was added to the culture at a MOI of 0.02. Reduction of the mixed culture at RT and 18°C was determined after seven hours and incubation overnight, while due to the slow growth of the bacteria at 15°C, 12°C, 9°C and 6°C, reductions were exclusively determined after incubation overnight. All experiments were carried out in triplicate.

### Analysis of bacteria that survived phage infection

The serotype of 100 colonies obtained from the 18°C infection trial was determined using specific PCR primers for O:3, O:9 and O:5,27 as previously published (Garzetti et al., 2014). Spot assays (as described above) were then employed to determine individual sensitivity to each of the five phages. The untreated strains were used as control.

One representative of each resistant pattern was inoculated eight times (once per day, followed by incubation overnight). The sensitivity of the isolates was tested after four and eight incubations by spot assays using the untreated strains as control again.

### Transmission electron microscopy (TEM)

CsCl-purified phages were investigated by TEM using the negative staining procedure with uranyl acetate (VWR International, Darmstadt, Germany) as described by Jäckel et al. (2017). Specimens were examined by TEM using a JEM-1010 (JEOL, Tokyo, Japan) at an 80 kV acceleration voltage (Hammerl et al., 2021a).

### Phage DNA preparation, sequencing, and genome analysis

Short-read, paired-end whole-genome sequencing (WGS) was conducted for *Yersinia* phage genome determination. Phage DNA isolated from proteinase K/SDS treated particles was use for DNA sequencing library preparation using the DNA Flex Library Preparation kit (Illumina, San Diego, CA, USA). WGS (2 × 150 cycles) was conducted on an Illumina NextSeq500 device, as previously described (Hammerl et al., 2021b). De novo assembly and coding sequence prediction were performed using the SPAdes algorithm within the Pathosystems Resource Integration Center (PATRIC) database (version 3.6.20), resulting in single contigs with a mean sequencing depth of > 150 (Hammerl et al., 2021b). Basic sequence comparison was carried out using the NCBI blast-suite (https://blast.ncbi.nlm.nih.gov/Blast.cgi). All software tools were used with default settings. Basic sequence analysis was conducted using DS Gene (version 2.5; Accelrys Inc., San Diego, CA, USA) (Hammerl et al., 2021a).

### Nucleotide sequence accession

The genomes of the *Y. enterocolitica* phages vB_YenM_P744, vB_YenM_P778 and vB_YenM_P8 were deposited in GenBank under the accession numbers PP693299, PP693300 and PP693301, respectively.

## Results

### The phages are myoviruses, which do not share any genomic similarity

**FIGURE 1:**
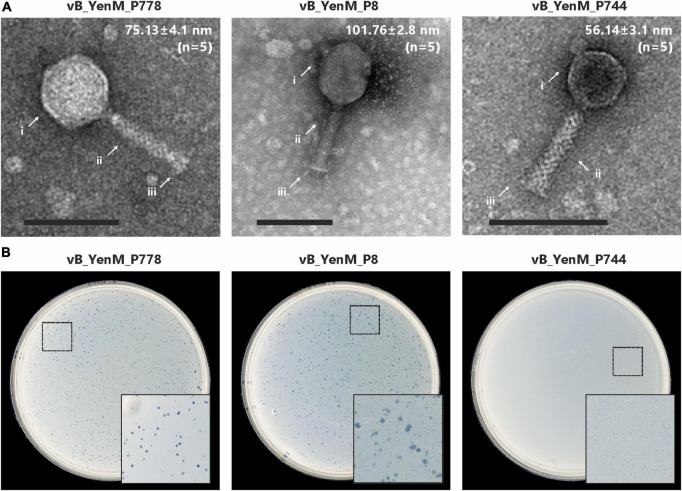
Transmission electron micrographs and plaque morphology of the *Y. enterocolitica* phages vB_YenM_788, vB_YenM_P8 and vB_YenM_744. **(A)** Morphology of phage particles. Mean head diameters are given for five measured virion particles of each phage. Magnification = 100,000 X, the bar represents 100 nm. Arrows indicate the head, tail and base plate of each myovirus. **(B)** Plaques, formed by the phages at room temperature. The position of the enlarged area of each plate is indicated.

**FIGURE 2:**
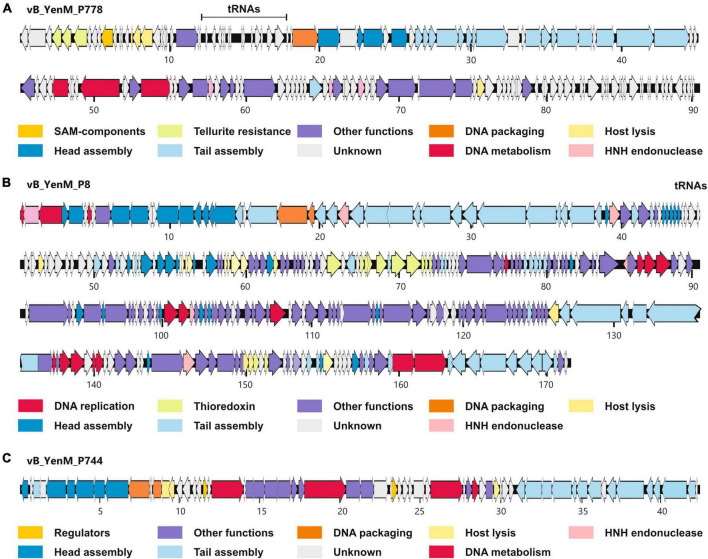
Gene maps of vB_YenM_788 **(A)**, vB_YenM_P8 **(B)** and vB_YenM_744 **(C)**. Putative genes are colored according to their predicted functions. The positions of tRNA genes are indicated. Numbers indicate kilobases of each genome. HNH, homing endonuclease.

At room temperature, phage vB_YenM_P8 and vB_YenM_P778 form rather small, clear plaques on susceptible *Y. enterocolitica* strains. The plaques of vB_YenM_P744 were much more turbid (Figure 1). At 30°C and 37°C, plaques were only observed with vB_YenM_P8. Electron micrographs revealed that the phages vB_YenM_P744, vB_YenM_P778 and vB_YenM_P8 are myoviruses with an isometric head and a contractile tail (Figure 1). However, while vB_YenM_P744 and vB_YenM_P778 possess an icosahedral head, the head of vB_YenM_P8 is prolate. The three phages also differ significantly regarding their genome size. vB_YenM_P744 has the smallest genome of 42,401 bp. By contrast, the genomes of vB_YenM_P778 (90,106 bp) and vB_YenM_P8 (171,477 bp) are much larger (Figure 2, Supplementary Table 1). Sequence analyses showed that vB_YenM_P8 is a T4-like phage. Its closest relative is *E. coli* phage UPEC07 (MW250787.1), to which it is 98.68% identical over 94% of their genomes. Significantly weaker identity values were determined to the closest related *Yersinia* phage PYps35T infecting *Y. pseudotuberculosis* (93% over 15% of the genomes) and to T4 (94% over 11% of the genomes) (Hammerl et al., 2021a). The great majority of the 309 predicted Open Reading Frames (ORFs) could functionally be assigned (Supplementary Table 1). There are more than 70 ORFs for virion assembly, but also a large number of ORFs probably involved in replication and recombination, e.g., six ORFs encoding homing endonucleases. In addition, the vB_YenM_P8 genome contains eleven ORFs for thioredoxin and two ORFs for tRNAs that may be important for DNA transcription and translation, respectively. Whereas vB_YenM_P8 is very similar to known phages, this does not apply to vB_YenM_P744 and vB_YenM_P778, which are novel phages (Figure 2). Phage vB_YenM_P778 is only very distantly related to some virulent *E. coli* and *Shigella* phages, e.g., JK55, to which it is 73% identical over 3% of the genomes. However, it is noteworthy that on its genome 20 tRNA-genes were identified for an optimized tRNA usage to supplement the host tRNA pool with self-encoded tRNAs corresponding to codons preferred in the phage genome, which may occur infrequently in the host (Albers and Czech, 2016). In addition, some resistance genes for tellurite, which is highly toxic for most bacteria (Nguyen et al., 2021). In addition, a number of ORFs probably encoding head and tail proteins and ORFs involved in nucleotide metabolism and phage-induced lysis were detected (Supplementary Table 1). The vB_YenM_P744 genome showed only some short stretches (less than 150 bp in length) that were similar to the chromosome of *Y. enterocolitica* strains. Here, for 44 out of 66 putative ORFs, functional assignments could be made, of which 21 ORFs may encode structural proteins (Supplementary Table 1). In addition, ORFs for the small and large subunit of the terminase, for cell lysis and for DNA replication were predicted. Genes for an integrase and repressor proteins could not be detected. However, as this phage formed turbid plaques on agar plates (Figure 1) and because some very short stretches of its genome showed a relationship to chromosomal sequences of *Y. enterocolitica*, we performed lysogenization experiments to elucidate whether it might be temperate. This was done by spotting a high-titer lysate of the phage onto a lawn of a susceptible strain followed by isolation of 20 colonies that survived the infection. Upon treatment with mitomycin C, none of them released an active phage, determined by spot assay.

### The host ranges of the phages complement each other well

To determine the host specificity of the phages, 53 strains belonging to the bioserotypes 4/O:3, 2/O:9 and 2/O:5,27, which cause most human infections in Europe, were tested (Hammerl et al., 2021b). In addition, eight 1B/O:8 strains were examined that occur only rarely in Europe. The study showed that vB_YenM_P744 revealed the broadest host range, as it lysed the 53 4/O:3, 2/O:9 and 2/O:5,27 strains with a high EOP (Table 1 and Supplementary Table 2). Phage vB_YenM_P778 lysed almost all 4/O:3 and 2/O:5,27 strains with a high EOP, but only two out of 19 strains belonging to the bioserotype 2/O:9. By contrast, the best results with vB_YenM_P8 were obtained with 2/O:9 strains, which were lysed at RT and 30°C with a high EOP. On the other hand, this phage infected only three out of 18 4/O:3 and 12 out of 16 2/O:5,27 strains with a rather low EOP. Thus, the three phages together lysed all 53 pathogenic *Y. enterocolitica* strains of the three bioserotypes. By contrast, 1B/O:8 strains were not lysed by any of the phages studied here (Table 1 and Supplementary Table 2).

**TABLE 1:** Host specificity of vB_YenM_P8, vB_YenM_P744 and vB_YenM_P778 and of the previously described phages vB_YenM_P281 and vB_YenP_Rambo at room temperature.

	vB_YenP_Rambo	vB_YenM_P281	vB_YenM_P778	vB_YenM_P8			vB_YenM_P744
**Bioserotype**	**RT**	**RT**	**RT**	**RT**	**28 °C**	**37 °C**	**RT**
4/O:3 (*n* = 18)	*n* = 5 (27.8%)	*n* = 18 (100%)	*n* = 18 (100%)	*n* = 3 (16.7%)	*n* = 4 (22.2%)	*n* = 0 (0%)	*n* = 18 (100%)
2/O:5,27 (*n* = 16)	*n* = 5 (31.3%)	*n* = 16 (100%)	*n* = 15 (93.8%)	*n* = 12 (75%)	*n* = 10 (62.5%)	*n* = 1 (6.3%)	*n* = 16 (100%)
2/O:9 (*n* = 19)	*n* = 19 (100%)	*n* = 19 (100%)	*n* = 2 (10.5%)	*n* = 19 (100%)	*n* = 16 (84.2%)	*n* = 7 (36.8%)	*n* = 19 (100%)
1B/O:8 (*n* = 8)	*n* = 0 (0%)	*n* = 2 (25%)	*n* = 0 (0%)	*n* = 0 (0%)	*n* = 0 (0%)	*n* = 0 (0%)	*n* = 0 (0%)

### The phages lyse their hosts also at low temperatures applying a low MOI

**FIGURE 3:**
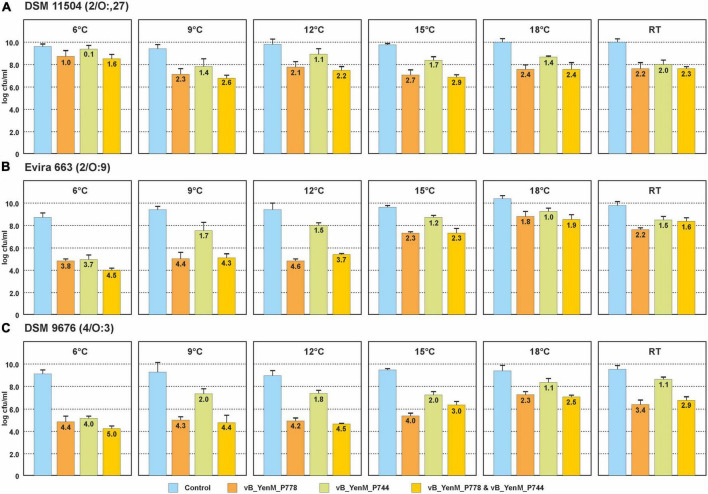
Reduction values of the 4/O:3 strain DSM 9676 **(A)**, 2/O:9 strain Evira 663 **(B)** and 2/O:5,27 strain DSM 11504 **(C)** by the phages vB_YenM_744 and/or vB_YenM_788 at temperatures between 6°C and RT. Means and standard deviations of three replicates are shown.

**FIGURE 4:**
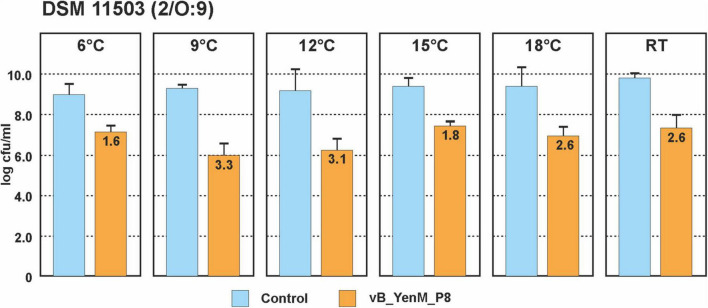
Reduction values of the 2/O:9 strain DSM 11503 by phage vB_YenM_P8 at temperatures between 6°C and RT. Means and standard deviations of three replicates are shown.

Since the three phages revealed a strong lytic activity at RT, we wanted to learn, whether they are able to lyse theirs hosts at low temperatures. Therefore, we chose one strain each of the bioserotypes 4/O:3, 2/O:5,27 and 2/O:9 and cultivated them at 6°C, 9°C, 12°C, 15°C, 18 °C and at RT. The strains were infected with vB_YenM_P744 and/or vB_YenM_P778, while due to the restricted lytic activity of vB_YenM_P8 on 4/O:3 and 2/O:5,27, only the 2/O:9 reference strain DSM-11503 was examined. Due to the slow growth of the bacteria at low temperatures, the infections were performed overnight using a MOI of 0.1. All samples containing phage revealed a significant lower optical density than the controls. The 4/O:3 strain alone grew well at all temperatures, even though below 15°C, slightly lower counts were determined (data not shown). Reductions between 2.3 and 4.4 log_10_ units were obtained with vB_YenM_P778, while vB_YenM_P744 reduced the strains by 1.1 to 4 log_10_ units (Figure 3). It is notable that with both phages the strongest reduction was observed at 6°C. A combination of the phages resulted in a reduction of even up to 5 log_10_ units at 6°C. Similar results were achieved with the 2/O:9 strain (Figure 3). Phage vB_YenM_P778 again reduced the bacteria more efficiently (1.8 to 4.6 log_10_ units) than vB_YenM_P744 (1 to 3.7 log_10_ units). Both phages together lead to a reduction of up to 4.5 log_10_ units at 6°C. Using the 2/O:5,27 strain, the results were slightly different. Here the strongest reduction by vB_YenM_P778 (2.7 log_10_ units) and by both phages (2.9 log_10_ units) were achieved at 15°C, while that by vB_YenM_P744 (2.0 log_10_ units) alone occurred at RT (Figure 3). Finally, also phage vB_YenM_P8 lysed the tested 2/O:9 strain efficiently at cold temperatures with a maximum reduction (3.3 log_10_ units) at 9°C (Figure 4). To ensure that bacteria were not lysed by the phages after plating on agar, we compared the numbers of colonies after incubation overnight at RT, 30°C, and 37°C, because none of the phages was able to lyse its respective host at 37°C. Almost identical numbers of colonies were identified at each temperature indicating that the incubation on agar plates did not falsify the obtained results. We also tested the plaque forming ability of the phages at low temperatures. While vB_YenM_P744 and vB_YenM_P778 formed plaques at 6°C after incubation for at least 72 h, the threshold temperature for plaque forming of vB_YenM_P8 was 15°C (Supplementary Figure 1).

### A mixture of *Y. enterocolitica* serotypes was significantly reduced by a five-phage cocktail

**FIGURE 5:**
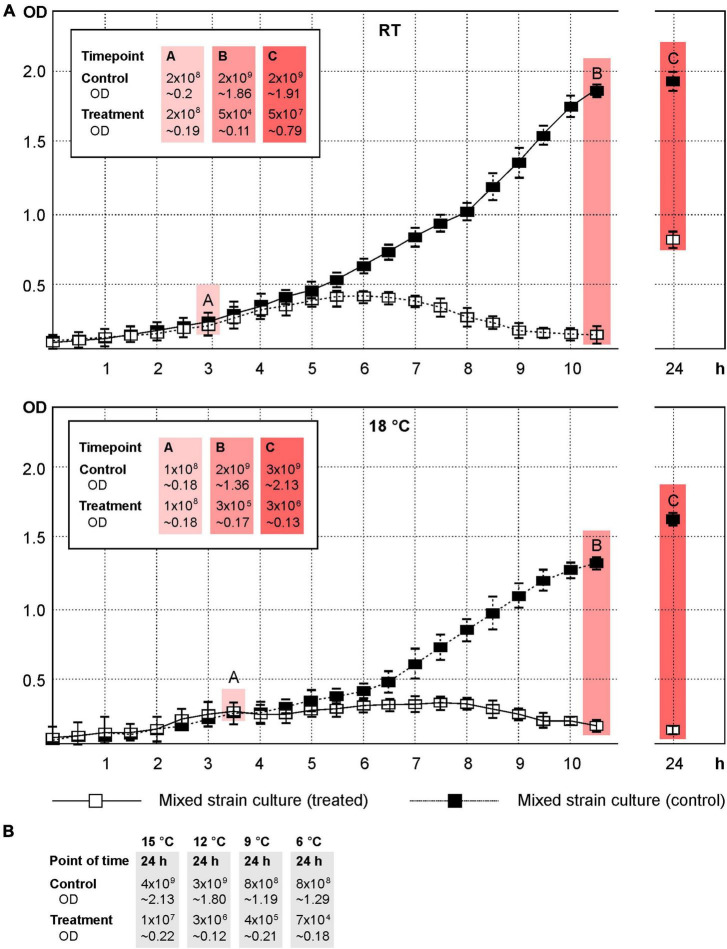
A mixture of the strains DSM 9676, DSM 11503 and DSM 11504 was efficiently lysed by the five phages at temperatures down to 6°C. Shown are the OD_588_ values of the cultures and the cfus. **(A)** Growth curves of the control and phage treated cultures at RT and 18°C. The points A, B and C indicate the time of adding the phages, first and second determination of cfu’s, respectively. **(B)** Reduction of the mixture of strains at 15°C, 12°C, 9°C and 6°C after incubation overnight.

The phages vB_YenM_P744, vB_YenM_P778 and vB_YenM_P8 lysed the important bioserotypes 4/O:3, 2/O:9 and 2/O:5,27 at low temperatures and thus complemented the lytic activity of the previously described phages vB_YenM_P281 and vB_YenP_Rambo, which are strongly lytic at 28°C and 37°C (Hammerl et al., 2021b). However, as the latter two phages also showed a good lytic activity at RT (Table 1), we wanted to learn, whether the five phages can efficiently reduce a mixture of the three bioserotypes at low temperatures and at a low MOI (0.1). In addition, we wanted to gather information about possible phage resistance. At RT the growth curve of the mixed culture showed a strong decrease in optical density starting already 90 min after adding the phages (Figure 5A). A very low optical density of the infected culture remained throughout the experiment, whereas that of the control increased constantly. After seven hours and incubation overnight, the numbers of bacteria were determined and reductions of approximately 4.5 log_10_ and 1.7 log_10_ units, respectively, were achieved. At 18°C, the results were very similar (Figure 5A). After seven hours, a reduction of almost 4 log_10_ units was obtained, while that after incubation overnight was approximately 2.5 log_10_ units. Compared to the initial number of bacteria (before phages were added), reductions of 2.3 and 1.1 log_10_ units were measured. Significant reductions were also determined at 15°C (2.3 log_10_ units), 12°C (3.0 log_10_ units), 9°C (3.2 log_10_ units) and 6°C (4.0 log_10_ units) after incubation overnight (Figure 5B).

To elucidate, which serotype survived phage infection, 100 colonies were picked from an agar plate incubated at RT and their serotype was determined using a serotype-specific PCR. Sixty O:9 colonies were isolated, whereas 32 and 8 colonies belonged to the serotype O:5,27 and O:3, respectively. The colonies and the untreated strains as control were then tested in terms of their resistance to the five phages by spot assays. While the control strains showed the typical lysis patterns with the five phages, all isolated colonies revealed resistance to at least one phage, but behaved quite diversely. Eight different resistance patterns were observed (Tables 2, 3 and Supplementary Table 3). However, some patterns were prevailing and there were some strong correlations between the serotype and the respective resistance pattern. Serotype O:3 revealed exclusively resistance to the phages vB_YenM_P744 and vB_YenP_Rambo, while all isolates of this serotype were sensitive to vB_YenM_P778 and vB_YenM_P281. On the other hand, most isolates of serotype O:5,27 were still lysed by three phages (vB_YenM_P778, vB_YenM_P281 and vB_YenP_Rambo) and showed a growth inhibition with vB_YenM_P744. Finally, the vast majority of O:9 isolates were lysed by vB_YenM_P8, whereas they were resistant to vB_YenM_P744, vB_YenM_P281 and vB_YenP_Rambo. Besides these predominant patterns, some individual patterns were obtained for single isolates (Table 2 and Supplementary Table 3). After four-time and eight-time inoculation of one representative of each pattern, phage resistance was examined again, and the same patterns were obtained as before suggesting that a reversion to the original phenotypes of the tested strains did not occur.

**TABLE 2:** Resistance patterns of the 100 *Y. enterocolitica* isolates that survived phage infection.

Resistance type	vB_YenP_Rambo	vB_YenM_P281	vB_YenM_P778	vB_YenM_P8	vB_YenM_744
A	−	+	+	n.a.	−
B	+	+	+	n.a.	GI
C	−	−	n.a.	+	−
D	+	+	n.a.	+	+
E	+	−	n.a.	+	−
F	+	−	n.a.	−	−
G	+	+	−	n.a.	−
H	+	+	n.a.	−	−

**TABLE 3:** Resistance patterns of the 100 *Y. enterocolitica* isolates that survived phage infection.

Resistance type	Serotype O:3 (*n* = 8)	Serotype O:5,27 (*n* = 32)	Serotype O:9 (*n* = 60)	Total (*n* = 100)
A	*n* = 8 (100%)	*n* = 13 (40.6%)	n.a.	*n* = 21 (21%)
B	n.a.	*n* = 17 (53.1%)	n.a.	*n* = 17 (17%)
C	n.a.	n.a.	*n* = 48 (80%)	*n* = 48 (48%)
D	n.a.	n.a.	*n* = 1 (1.7%)	*n* = 1 (1%)
E	n.a.	n.a.	*n* = 5 (8.3%)	*n* = 5 (5%)
F	n.a.	n.a.	*n* = 3 (5%)	*n* = 3 (3%)
G	n.a.	n.a.	*n* = 3 (5%)	*n* = 3 (3%)
H	n.a.	*n* = 2 (6.3%)	n.a.	*n* = 2 (2%)

n.a., not applicable; −, no plaque formation; +, plaque formation; GI, growth inhibition.

## Discussion

Phages are a promising tool to combat pathogenic bacteria along the food chain. They can be either applied in living animals (pre-harvest) or during food processing (post-harvest). However, whereas e.g., in the gut of many animals, phages encounter and lyse their host cells at a temperature of approximately 37°C, much lower temperatures prevail mostly during the following steps of food production. Thus, phages intended for applications should meet the respective requirements, which means that they should exhibit a good lytic activity at low temperatures, when applied during food production. The three myoviruses described here revealed a broad host range (between 54.1% and 88.9% of 61 tested pathogenic *Y. enterocolitica* strains were lysed). In addition, they showed a good lytic activity between 6°C and RT, whereas at 30°C and 37°C, only vB_YenM_P8 revealed lysis of some 2/O:9 and 2/O:5,27 strains. This phage is related to T4-like phages and similar to the *Y. enterocolitica* phages èR1-RT and TG1, which, however, were reported to lyse several serotypes like O:3, O:9 and O:5,27 only at or below 25°C (Leon-Velarde et al., 2016). Though, it is interesting that in the cited study the proportion of phage sensitive O:3 strains was also lower than that of O:9 and O:5,27. Whether in vB_YenM_P8, like in èR1-RT and TG1, OmpF is a receptor for the phage that is strongly repressed at 37°C, is yet not clear, since a significant number of O:9 strains were lysed by this phage at 37°C. Unlike vB_YenM_P8, vB_YenM_P744 and vB_YenM_P778 did not show any significant similarity to known phages. Both were isolated from fecal samples of wild boars, which raises the question whether they may be active in the gut of those animals where much higher temperatures than RT occur. However, it cannot be excluded that at 37°C, the phages can also infect other host bacteria, in which they can propagate to high numbers. Another *Y. enterocolitica* phage, which is exclusively lytic at RT, is phage vB_YenS_P400, a siphovirus, which, however, has an extremely narrow host range only infecting two 4/O:3 strains out of 80 tested *Yersinia* strains belonging to various species (Hammerl et al., 2022a). Similarly, phage phi 80-18 was shown to infect only 16 out of 115 *Yersinia* strains. It specifically lysed several serotypes of biotype 1B and some biotype 1A strains, whereby at 4°C and 28°C, much better propagation was determined than at 37°C (Filik et al., 2020). Jun et al. (2018) reported on experiments with several food samples (raw pork, ready-to-eat pork, milk) that were spiked with an O:9 strain (10^3^ CFU/g or ml) and afterwards treated with a èR1-RT and TG1 related phage (fHe-Yen9-01) at a MOI of 10^5^ (approximately 2 x 10^8^ PFU/g or ml) (Jun et al., 2018). They found that the phage could decrease the number of bacteria on raw pork (4°C, 72 h), ready-to-eat pork (26°C, 12 h), and milk (4°C, 72 h) by 1 to 3 logs from the original levels of 2-4 × 10^3^ CFU/g or ml. The experiments were conducted at 4°C (raw pork and milk) for 72 h or at 26°C (ready-to-eat pork) for 12 h. In another study, the lytic potential of two *Y. enterocolitica* broad host range phages (vB_YenM_P281 and vB_YenP_Rambo) was studied. These two phages caused the strongest lysis of their hosts at 37°C, but were also able to lyse them at 17°C, when a MOI of 2 was applied (Hammerl et al., 2021b). We therefore examined the lytic activity of vB_YenM_P281 and vB_YenP_Rambo in combination with the three phages described here on a mixture of *Y. enterocolitica* strains and found a strong reduction of the bacteria at RT and 18°C after seven hours (4 to 4.5 log_10_ units) and also after incubation overnight (1.7 to 3 log_10_ units). Moreover, at lower temperatures down to 6°C, even stronger reductions (up to 4.1 log_10_ units) were determined after incubation overnight. Thus, the cocktail is well suited to reduce *Y. enterocolitica* at low temperatures, at which this psychrophilic agent can proliferate. The fact that the cultures did not show regrowth after this time period suggests only a low rate of phage resistance to the cocktail. Indeed, testing of 100 colonies that had survived the infection revealed sensitivity to at least one phage of the cocktail. Moreover, bioserotype 4/O:3, which causes in Europe most human infections, revealed much less resistance than bioserotype 2/O:9. Thus, the cocktail is best suited if bioserotype 4/O:3 prevails. It is very likely that the isolated bacteria survived the infection, because they did not encounter the respective phage particle or that they reached the stationary growth phage, where phage infection is limited. The observed resistances remained stable during cultivation of the isolates It indicates that mechanisms like phase switching, transcriptional regulation, or asynchronic proliferation of cells expected to be reversible did not occur here. Instead, mutations resulting in a high and not reversible resistance rate are more likely. As the five phages do not show any genetic relationship to each other and as they also deviate regarding their host specificity at different temperatures, it can be suggested that they do not all use the same host receptor for binding. These features of the cocktail are very important for usage, because the phages are able to lyse a broad spectrum of *Y. enterocolitica* strains and resistance, which is often associated with prevented binding to the bacterial cell, cannot easily occur as with closely related phages. It makes the cocktail very promising for applications. We will now examine its lytic activity on food samples like pig skin.

## Data availability statement

The datasets presented in this study can be found in online repositories. The names of the repository/repositories and accession number(s) can be found here: https://www.ncbi.nlm.nih.gov/genbank/, PP693299, PP693300 and PP693301.

## Author contributions

JH: Conceptualization, Data curation, Formal analysis, Funding acquisition, Investigation, Project administration, Software, Validation, Visualization, Writing–original draft, Writing–review and editing. MP: Investigation, Writing–review and editing, Conceptualization. SE-A: Investigation, Writing–review and editing. DM: Investigation, Writing–review and editing. CJ: Data curation, Formal analysis, Investigation, Writing–review and editing. SH: Conceptualization, Funding acquisition, Investigation, Project administration, Supervision, Writing–original draft, Resources, Validation.

## Footnotes

1
https://lpsn.dsmz.de/genus/yersinia

## Supplementary material

Supplementary Figure 1 Plaques formed by the three phages at 6 C (vB_YenM_788 and vB_YenM_744) and 15 C (vB_YenM_P8).

Supplementary Table 1 ORF analysis of vB_YenM_788, vB_YenM_P8 and vB_YenM_744.

Supplementary Table 2 *Y. enterocolitica* strains used in this study for the determination of the host range of the phages. Note: ++++++, strong lytic activity (single plaques obtained with 10^4^ and 10^5^ dilution), ++++, medium lytic activity (single plaques obtained with 10^2^ and 10^3^ dilution), ++, weak lytic activity (single plaques obtained with 10^−1^ dilution or an undiluted lysate), −, no lytic activity, GI, growth inhibition.

Supplementary Table 3 Phage resistance of the *Y. enterocolitica* 4/O:3, 2/O:9 and 2/O:5,27 isolates to the five phages.
