# Supplementary figures and images for: Biological and genomic characterization of three psychrophilic Y. enterocolitica phages
Source: Front Microbiol. 2024 Jul 11;15:1423610. doi: 10.3389/fmicb.2024.1423610 (PMC11269248; doi:10.3389/fmicb.2024.1423610)

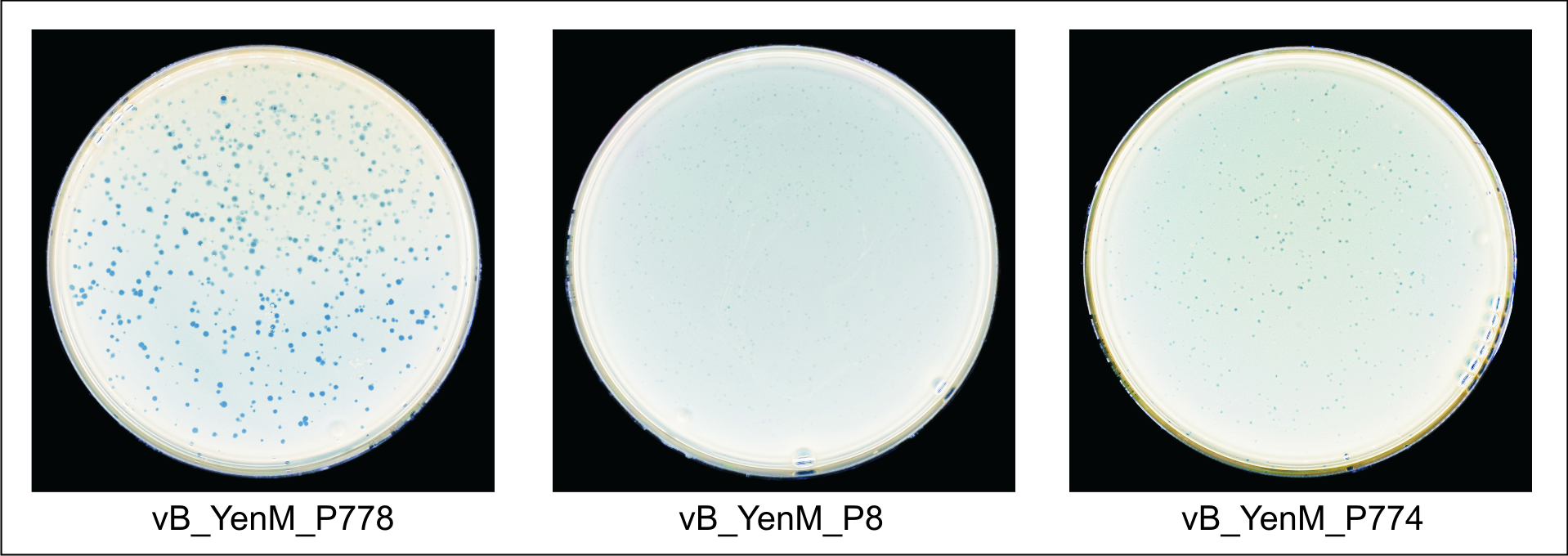

Supplement: Supplementary Figure 1 — Plaques formed by the three phages at 6 C (vB_YenM_788 and vB_YenM_744) and 15 C (vB_YenM_P8). [file Image_1.TIF]
